# Prevalence and associated factors of depression among tuberculosis patients in Eastern Ethiopia

**DOI:** 10.1186/s12888-019-2042-6

**Published:** 2019-03-01

**Authors:** Tamirat Tesfaye Dasa, Aklilu Abrham Roba, Fitsum Weldegebreal, Frehiwot Mesfin, Abiyot Asfaw, Habtamu Mitiku, Zelalem Teklemariam, Bahubali Jinnappa Geddugol, Mahantash Naganuri, Hilina Befikadu, Eden Tesfaye

**Affiliations:** 1 Haramaya University, College of Health and Medical Sciences, P.O.Box-235, Harar, Ethiopia; 2 Haramaya University, College of Social Sciences and Humanities, P.O.Box-138, Dire Dawa, Ethiopia; 3 Haramaya University, College of Natural and Computational Sciences, P.O.Box-138, Dire Dawa, Ethiopia

**Keywords:** Depression, MDR/ TB patients, Comorbidity, Stress, Anxiety, Eastern Ethiopia

## Abstract

**Background:**

Depression among tuberculosis patients, especially in settings with low economic status is common. Screening for depression in all levels of health facilities can identify patients who need support and treatment for depression.

**Objective:**

The aim of this study was to assess the prevalence and associated factors of depression among tuberculosis patients in Eastern Ethiopia.

**Methods:**

An institutional based cross-sectional study was conducted among 403 tuberculosis patients attending in eleven tuberculosis treatment centers in eastern Ethiopia from February to July 2017. Depression was measured using the Patient Health Questionnaire. Data was collected consecutively until the required sample size was obtained. Tuberclusis  patients who were under anti tuberculosis treatments for more than one month were included. Data were analyzed with Statistical Package for Social Sciences (SPSS) version 20. Bivariate and multivariate logistic regression models were applied to identify independent factors for dependent variable depression and *P*-values < 0.05 considered statistically significant.

**Results:**

A total of 403 tuberculosis patients were included in the study. The prevalence of depression among tuberculosis patients was 51.9% (95%CI = 42.7, 62.2%) with 34.2% were mild cases. In our logistic regression analysis, odds of developing depression among tuberculosis patients with age less than 25 years were 0.5(50% protective effect) [AOR = 0.5, 95% CI 0.26–0.99] where as patients with a monthly income within the 25^th^percentile were four times higher odds to have depression [AOR = 3.98, 95% CI: 2.15–7.39].

**Conclusion:**

The prevalence of depression was high in this study. Age, low monthly income, the category of patients as “new tuberculosis treatment” and the first 3 months of treatment was associated with depression among tuberculosis patients. Health facilities should integrating mental health services with tuberculosis clinics, especially assessing and treating TB patients for depression, is vital.

## Background

Depressionis a common mental disorder characterized by sadness, loss of interest or pleasure, feelings of guilt or low self-worth, disturbed sleep or appetite, feelings of tiredness, and poor concentration. It can be long-lasting or recurrent, substantially impairing an individual’s ability to cope with daily life and can also lead to suicide. At a global level, over 300 million people are estimated to suffer from depression, equivalent to 4.4% of the world’s population. In Ethiopia, a total of 4,480,113 people are estimated to suffer from depression, which is equivalent to 4.7% the total population [1]. The problem is highly reported from eastern Ethiopia. In one study, the magnitude depression was 14.9% among the adult population in Harari region, eastern Ethiopia [2].

People with tuberculosis (TB) are often suffer from depression [3, 4].The prevalence of depression among TB patients was reported variable from different studies which is in Nigeria(41.1%) [5], Cameron (61.1%) [6] andPakistan (56%) [7]. InEthiopia, the prevalence depression among TB patients was reported as 43.4% from Wolaita Sodo, South Ethiopia and 54% different areasSouth Ethiopia (13).Depression is more common in Multi drug resistant TB patients than other pulmonary TB patients [8].

Depression weakens the psychosocial welfare and results in negative treatment outcomes among TB patients [9, 10]. It can also negatively affects health-related quality of life of TB of patients [11]. In addition, TB patients with depressive symptoms have reduce social contact and ignore social responsibilities especially at the stage of coughing that leads to low self-esteem and hopelessness [12].

Several factors were associated with the occurrence of depression among TB patients. Human Immune deficiency Virus (HIV) infection, poor social support and perceived stigma have a higher risk of developing depression among TB patients [13–15]. Other risk factorslike side effects of the drugs, and the financial constraints, Older age, female sex, duration of illness, level of education were also identified [16, 17].

There was high prevalence report of TB from presumptive TB patients attending in Ethiopia [18]. However, there is a dearth of information on the comorbidity of depression with tuberculosis in eastern part of Ethiopia. Therefore, this study was tried to assess the magnitude of depression and associated factors among TB patients in eastern Ethiopia.

## Methods and materials

### Study setting

This study was conducted in eight hospitals and three health centers in eastern Ethiopia including Jugal, Hiwot Fana, Karamara, Dil Chora, Sabian, Haramaya, Deder and Chiro hospitals. The health centers were Number One, Legahare and Amir Nur. The study area has an urban, semi-urban and rural setting with diverse geo-climatic conditions that incorporate low, mid and high lands, and peoples with diverse lifestyles including farmers, agro-pastoralists, pastoralists and urban dwellers.

### Study design and period

An institution-based cross-sectional study was conducted from February to July 2017.

### Study population

Four hundred and three TB patients who were under anti TB treatments for more than one month (after started medication for month) were participated in this study. They have communication problems and had completed treatment were excluded.

### Sample size and sampling technique

The sample size calculated by Open Epi version 3 online software with the following assumptions: population size (for finite population correction factor or fpc) (N) =1000,000, hypothesized frequency of outcome factor in the population (p) =54+/− 5 [17], margin of error 5, 95% CI, and non-response rate = 5%. The final calculated sample size including 5% non-response rate was 403 TB patients. Study participants were proportionally allocated for each health facilities according to patients flow by referred the previous year annual reports. TB patients that fulfilled the inclusion criteria were included in the study by consecutively. TB patients that fulfilled the inclusion criteria were selected by consecutive sampling techniques. All eligible TB patients were included to participate in the study until the required sample size obtained.

### Study variables

Presence of depression among TB patients was our dependent variable. The independent variables included age, sex, residence, religion, ethnicity, marital status, occupational status, educational status, family size, TB treatment duration, type of medication, level of TB treatment and quality of life.

#### Operational definition

Percentile of income = (number of people behind of your income /total number of people in study income) × 100. That means (25^th^ percentile can be defined as the lowest income score that is greater than 25% of the income, for others like 50^th^ and 75^th^).

### Data collection

Data was collected in the local language of the patients, using face-to-face interviews, by 8 psychiatric nurses, in outpatient TB clinics, after informed voluntary and signed consent were obtained. Depression was measured using the Patient Health Questionnaire (PHQ-9) and patients with a cut-off value of five or more [19] considered to have a depressive disorder.

### Data collection instruments

Data was collected by structured interview questionnaire and it has two parts. The 1st part contains: sociodemographic characteristics of participants and clinical conditions; the 2nd part contains Patient Health Questionnaire-9 (PHQ-9) depression scale. Depression was assessed using (PHQ-9), questionnairewas prepared in English and translated to local languages (Amharic and Afan Oromo). (PHQ-9) scale has been formally validated in Ethiopia in threedifferent studiesat different setting [20, 21]. Measured using the score from PHQ-9, a nine-item depression-screening instrument that asks about the frequency of symptoms of depression in the past 2 weeks [11, 22]. Response categories of “not at all,” “several days,” “more than half the days,” and “nearly every day” are given a score of 0 to 3. Summary scores ranged from 0 to 27. Depression was defined using a score of 10 or higher, a well-validated cut point used in health care settings [22].

### Data processing and analysis

Data entered into Epi Data software version 3.1 and exported to SPSS version 20 for analysis. Descriptive statistics were used to describe the frequency, median, mean and standard deviation. The prevalence of depression was defined as the proportion of those TB patients with depression. Bivariate and multivariate logistic regression models were applied to identify independent factors for dependent variable, depression and *P*-values < 0.05 considered statistically significant.

## Results

### Socio-demographic characteristics

A total of 403 TB patients participated in this study with response rate of 100%. Median age of the study participants was 30 years with a range of 7–74 yearsand with interquartile range of 18 years. Majority of TB patients were young adults and adolescents in age group less than 35 years (61.5%), Urban dweller (70.2%), married (53.6%) males in sex (59.3%) and without any formal education and attended only primary schools were (61.3%) and with family size. 67% (67%) of TB patients were newly diagnosed and 70% TB patients were on the treatment for more than 3 months (13.6%) were HIV positive.The prevalence among MDR-TB patients among TB patients was 24.8% (Table 1).

**Table 1:** Socio-demographic characteristics of participants attending TB clinics in health facilities of Eastern Ethiopia, 2017 (*n* = 403)

Characteristics		No.	%
Age	≤24	125	31.0
	25–34	123	30.5
	≥35	155	38.5
Gender	Male	239	59.3
	Female	162	40.7
Residence	Urban	283	70.2
	Rural	120	29.8
Marital status	Married	216	53.6
	Single	147	36.5
	Separated / Widowed	40	9.9
Educational status	No formal education	104	25.8
	Primary education	143	35.5
	Secondary and above	156	38.7
Occupations	Employed	56	13.9
	Private	131	32.5
	Student	69	17.1
	Farmer/Daily laborers	147	36.5
Family size	1–2	90	22.3
	3–5	205	50.9
	More than 5	108	26.8
Monthly Income	25^th^ percentile	124	30.8
	50^th^ percentile	109	27.0
	75^th^ percentile	170	42.2
Level of TB treatment	New treatment TB	272	67.5
	Retreatment and MDR- TB	131	32.5
TB treatment duration	< 3 months	119	29.5
	3–6 months	215	53.3
	> 6 months	69	17.1

### Magnitude of depression

The prevalence of depression in people with TB using a cut-off value of five and above was 51.9% (95%CI = 42.7, 62.2%) (Table 2).

**Table 2:** Depression scoring scale among in TB patients among Eastern Ethiopia, 2017(*n* = 403)

Depression Categories	Scoring	Frequency	Percent (%)	95% Confidence Interval
No depression	0–4	194	48.1	[43.4, 52.9]
Mild depression	5–9	138	34.2	[30,38.7]
Moderate depression	10–14	60	14.9	[11.7,18.6]
Moderately severe depression	15–19	10	2.5	[1,4.2]
Severe depression	20–27	1	–	[0.0,0.7]
Total		403	100	

### Factors associated with depression among TB patients

In Multivariate logistic regression analysis, we found out statistically significant associations between dependent and independent variables. Among tuberculosis patients age group less than 25 years, the odds of developing depression were 50% lower than those older than 35 years (50% protective effect not to develop depression) [AOR = 0.5, 95% CI 0.26–0.99]. Whereas patients with a monthly income fall in the 25th percentile were four times higher odds to have depression [AOR = 3.98, 95% CI: 2.15–7.39].Similarly, TB patients within the 25th to 50th percentile were twice as likely to develop depression [1.93, 95th CI 1.08–3.44) (Table 3).

**Table 3:** Socio-demographic factors associated with depression among TB patients (AOR with 95% CI) in eastern Ethiopia from February to July 2017 (*n* = 403)

Variables	Categories	Depression		COR (95% CI)	P-Value	AOR (95% CI)	P-Value
		Yes	No				
Age	≤24	52	73	0.45 (0.28–0.73)	**0.001**	0.5 (0.26–0.99)	0.047*
	25–34	62	61	0.64 (0.40–1.04)	0.07	0.58 (0.335–0.92)	0.05*
	≥35	95	60	1		1	
Residence	Urban	136	147	1.68 (1.09–2.59)	0.019	0.82 (0.47–1.43)	0.11
	Rural	73	47	1		1	
Marital status	Married	125	91	1.52(0.77–2.99)	0.22	2 (0.93–4.4)	0.28
	Single	65	82	0.88(0.44–1.77)	0.71	1.8 (0.77–3.49)	0.076
	Separated / Widowed	19	21	1		1	
Educational status	No formal education	63	41	1.61(0.98–2.67)	0.06	0.69 (0.36–1.34)	0.08
	Primary education	70	73	1.01(0.64–1,59)	0.97	0.81 (0.47–1.39)	0.99
	Secondary and above	76	80	1		1	
Occupations	Gov’t Employee/ NGO	29	27	0.61(0.33–1.13)	0.12	1.0 (0.46-2.29)	0.15
	Private/merchant	65	66	0.56(0.34–0.90)	0.02	0.72(0.39-1.31)	0.06
	Student	21	48	0.25(0.13–0.46)	**0.000**	0.25 (0.11–0.57)	0.04*
	Farmer/ Housewife/ Daily laborers	94	53	1		1	
Family size	1–2	40	50	0.60(0.34–1.04)	0.70	0.66 (0.34–1.3)	0.12
	3–5	107	98	0.81(0.51–1.30)	0.38	1.0 (0.6–1.8)	0.29
	More than 5	62	46	1		1	
Monthly Income	25th percentile	80	44	2.42(1.50–3.89)	**0.000**	3.98 (2.15–7.39)	0.000*
	50th percentile	56	53	1.40(0.87–2.28)	0.17	1.93 (1.08–3.44)	0.026*
	75th percentile	73	97	**1**		**1**	
Level of TB treatment	New treatment TB	134	138	0.73(0.48–1.10)	**0.13**	0.442 (0.24–0.83)	0.010*
	Retreatment/MDR TB	75	56	1		**1**	
HIV status	HIV positive	28	27	0.96(0.54–1.69)	0.87	0.99 (0.51–1.92)	0.92
	HIV negative	181	167	1		1	
TB treatment duration	< 3 months	74	45	1.60(0.88–2.91)	0.13	3.86 (1.6–9.3)	0.003*
	3–6 months	100	115	0.85(.49–1.45)	0.54	1.57 (0.72–3.4)	0.58
	> 6 months	35	34	1		1	

N: B:) *indicates a *p*-value < 0.05 which is statistically significant

## Discussions

There were very limited studies that assessed the burden of depression among TB patients globally. When compared to the available evidences, magnitude of depression in our study is comparable to most of sub-Saharan African studies but higher than other settings. This study revealed that 51.9% of TB patients have probable depression. The finding was comparable with other studies carried out in southern Ethiopia such as 43.4% in Wolayta zone [23], and 54% Gurage and Silte zone [24]. It is also similar with other studies conducted sub-Saharan Africa 49.4% in Angola [25] and 61.1% findings of the Southwest Region of Cameroon [26]. The prevalence of depression among TB patients in this study is slightly higher than other similar studies elsewhere like 35% in India [27], 19% in Turkey [28].

Severity of depression ranges from a mild form that may not need any medical treatment to the most severe presentations that may require thorough assessment and appropriate management. In this study, almost half of the TB patients were presented with mild to moderate depression while 2.7% have severe form that requires appropriate treatments. Diagnosing the severity may be important because individuals with advanced forms of depressions may be less likely to adhere to anti-TB drugs which increased risk of drug-resistance [11, 29], poorer quality of life and greater disability [11], lack of adherence to anti-TB treatment and Poor treatment outcomes including death.

In this study, age was found to be one of the risk factors for depression. As similar to another Ethiopian study [17], age less than 35 years was protective for depression among TB patients. This may be at younger age people may engage in different activities to earn money which may increase social interaction and most probably get support from colleagues or relatives. At older age, life in poor countries may be challenging as the habit of saving was low, engagement to economical activities may be stressful beside the challenges of tuberculosis like stigma, discrimination, anti-Tb side effects [30]. These stressful life events and chronicity of tuberculosis were associated with depression in studies conducted in Nigeria and Ethiopia [17, 31].

Low monthly income and depression were significantly associated in this study. Low income in TB patients has the adverse effect on depression. This is similar to a study conducted in China [32], South Africa [33], shanty towns in Lima [34]. In settings with a high burden of tuberculosis, the low income generated will result in difficulty of covering costs for treatment, even if the anti-TB drugs are provided freely. The expenses for additional nutritional needs, transportation and missed work days due to fatigue, chest pain and symptoms of tuberculosis result in lower earnings, and lower earnings will result in psychological distress due to the inability to satisfy the demands of the individual and their household [35].

We found that newly diagnosed patients for TB were associated with depression (aOR = 0.39 (0.21–0.74). This is similar to a study in Lima, capital city of Peru, in which depression was highly prevalent among newly diagnosed TB patients [34]. This may be associated with the sign and symptoms of TB especially during the first 3 months in which patients are not familiar with the condition and psychological interventions are almost absent [17]. Another study indicated that being on retreatment for TB (aOR = 11.2, 95% CI: 5.2–31.1, *P* < 0.001) and having discontinued treatment (aOR = 8.2, 95% CI: 1.1–23.3, *P* < 0.05) were factors associated with having a higher chance of being depressed [36].

Tuberculosis patients are challenged by mental health problems too. It is difficult to them to lead socially and economically productive life with the health status currently they have due to social isolation [37], depression and an enormous economic burden [38–40]. Therefore, programs designed to control/eliminate tuberculosis in community setting at local, national or international level should also screen and manage depression in addition to financial and social support. For patients admitted to health institutions due to tuberculosis or its complication, it’s better to screen for depression and treat it. This may improve treatment outcome and play a positive role in effort to control and eliminate tuberculosis.

Some limitations associated with this study include missing of some important variables not included in PHQ-9 tools such as substance use and smoking which are found to be associated with depression. From co-morbid diseases, only HIV was assessed. On the other hand, the included patients were at any stage of follow-up rather than a specific point in treatment in which true prevalence may be masked by the effect of treatment.

## Conclusion

The burden of depression among tuberculosis patient is high (51.9%). Financial constraint, older age and new TB patients are risk to develop depression. Depression should be assessed in all TB treatment centres and health professionals should provide appropriate psychological and medical treatment.

## Abbreviations

CI: Confidence Interval
DM: Diabetes Mellitus
HIV: Human Immune deficiency Virus
MDR/TB: Multidrug Resistant Tuberculosis
PHQ: Patients Health questionnaire
TB: Tuberculosis
WHO: world health organization
